# Role of stress-related hormones in plant defence during early infection of the cyst nematode *Heterodera schachtii* in Arabidopsis

**DOI:** 10.1111/nph.13395

**Published:** 2015-03-30

**Authors:** Nina Kammerhofer, Zoran Radakovic, Jully M A Regis, Petre Dobrev, Radomira Vankova, Florian M W Grundler, Shahid Siddique, Julia Hofmann, Krzysztof Wieczorek

**Affiliations:** 1 Division of Plant Protection, Department of Crop Sciences, University of Natural Resources and Life Sciences, UFT TullnKonrad Lorenz Str. 24, 3430, Tulln, Austria; 2 Institute of Crop Science and Resource Conservation, Department Molecular Phytomedicine, University BonnKarlrobert-Kreiten-Str. 13, D-53115, Bonn, Germany; 3 Institute of Experimental Botany, Academy of Sciences of the Czech RepublicRozvojová 263, 165 02, Prague 6, Lysolaje, Czech Republic

**Keywords:** defence responses, early infection, ethylene, *Heterodera schachtii*, jasmonic acid, plant-parasitic nematodes, salicylic acid

## Abstract

- *Heterodera schachtii*, a plant-parasitic cyst nematode, invades host roots and induces a specific syncytial feeding structure, from which it withdraws all required nutrients, causing severe yield losses. The system *H. schachtii*–Arabidopsis is an excellent research model for investigating plant defence mechanisms. Such responses are suppressed in well-established syncytia, whereas they are induced during early parasitism. However, the mechanisms by which the defence responses are modulated and the role of phytohormones are largely unknown.
- The aim of this study was to elucidate the role of hormone-based defence responses at the onset of nematode infection. First, concentrations of main phytohormones were quantified and the expression of several hormone-related genes was analysed using quantitative real-time (qRT)-PCR or GeneChip. Further, the effects of individual hormones were evaluated via nematode attraction and infection assays using plants with altered endogenous hormone concentrations.
- Our results suggest a pivotal and positive role for ethylene during nematode attraction, whereas jasmonic acid triggers early defence responses against *H. schachtii*. Salicylic acid seems to be a negative regulator during later syncytium and female development.
- We conclude that nematodes are able to impose specific changes in hormone pools, thus modulating hormone-based defence and signal transduction in strict dependence on their parasitism stage.

## Introduction

Sedentary plant-parasitic nematodes are responsible for severe economic and agricultural losses (Abad & Williamson, 2010), whereupon cyst nematodes (CNs) have a pivotal impact on wheat, soybean, potato and sugar beet production. In soil, host root exudates activate the second stage juvenile (J2) from dormancy in the egg shell and attract it to the root. There, it invades the cortex with the aid of its stylet and migrates intracellularly towards the vascular cylinder. It selects and pierces a single cell and releases saliva, triggering a series of cellular changes, such as increased cytoplasm streaming, enlargement of the nucleus, proliferation of plastids and mitochondria as well as dissolution of cell walls towards adjacent cells (Golinowski *et al*., 1996; Wyss, 1997). Fusion of this initial cell with neighbouring cells leads to the formation of a syncytium that induces as a strong sink within the plant, supporting the nematode with all required nutrients (Golinowski *et al*., 1996; Grundler *et al*., 1998; Jung & Wyss, 1999). The sugar beet CN *Heterodera schachtii* is one of the economically most important species, which is able to invade the model plant Arabidopsis, thus providing a powerful system to investigate plant–nematode interactions under laboratory conditions (Sijmons *et al*., 1991).

Plants have developed a battery of defence reactions in response to pathogen attack. In turn, nematodes evolved sophisticated mechanisms against pre-existing and inducible plant defence to reduce host cell damage and promote feeding site development (Wubben *et al*., 2008). Most studies available to date have concentrated on the later stages of nematode parasitism, when the syncytium is already well established and plant defence is widely suppressed (reviewed in Goverse & Smant, 2014; Ithal *et al*., 2007; Szakasits *et al*., 2009). Both migration and syncytium induction, however, are critical phases determining the success of the parasite. Until now, only a few studies have focused on the induction of plant defence and stress response during these phases. For instance, activation of defence signalling was observed in whole soybean (Alkharouf *et al*., 2006; Ithal *et al*., 2007; Mazarei *et al*., 2011) and Arabidopsis roots (Puthoff *et al*., 2003) infected with the CNs *Heterodera glycines* and *H. schachtii*, respectively. This inducible defence often depends on tissue-specific concentrations of different phytohormones (Denance *et al*., 2013), which, amongst other functions, trigger and coordinate responses to biotic stresses (Kunkel & Brooks, 2002; Farmer *et al*., 2003; Pieterse *et al*., 2009; Goverse & Bird, 2011). For instance, salicylic acid (SA) is a key component in plant defence against biotrophic pathogens (Pieterse *et al*., 2009), whereas the jasmonic acid/ethylene (JA/ET) pathway acts mainly against necrotrophic pathogens and herbivores (Staswick *et al*., 1998; Vijayan *et al*., 1998; Pieterse *et al*., 2009). Recently, auxins (e.g. IAA) and cytokinins, growth-promoting hormones, were also shown to be involved in the biotic stress responses (Mauch-Mani & Mauch, 2005; Kazan & Manners, 2009; Fu & Wang, 2011). The characteristic feature of phytohormones is their intensive cross-talk, which may be synergistic or antagonistic. Generally synergistic interaction has been observed between JA and ET in the activation of defence against wounding and necrotrophs, whereas competing defence pathways regulated mainly by SA and JA/ET are considered as mutually antagonistic (Glazebrook, 2005). On the one hand, hormone-dependent defence pathways, especially SA, ET and JA, play an important role in defence responses of the host plant to both root-knot nematodes (RKNs) and CNs (Wubben *et al*., 2001, 2008; Lohar *et al*., 2004; Ithal *et al*., 2007; Kyndt *et al*., 2012; Ali *et al*., 2013). On the other hand, several studies have shown a positive involvement of plant hormones during nematode feeding site formation (reviewed in Goverse & Bird, 2011; and in Kyndt *et al*., 2013). In particular, IAA and ET were found to be indispensable for initiation and proper feeding site development (reviewed in Gutjahr & Paszkowski, 2009; and in Goverse & Bird, 2011; Goverse *et al*., 2000; Karczmarek *et al*., 2004; Wang *et al*., 2007; Grunewald *et al*., 2009; Swiecicka *et al*., 2009). Further, gibberellins (GAs) have been suggested to maintain nematode feeding sites and act in their maturation (Klink *et al*., 2007; Kyndt *et al*., 2012).

Taken together, the different roles of phytohormones vary considerably during feeding site and nematode development, depending on the host, the nematode species and the stage of parasitism. However, their function in mediating attraction and host finding, as well as during early plant defence, is widely unexplored. Therefore, the aim of this work was to elucidate hormone-related defence pathways induced at the onset of nematode parasitism, including attraction, migration and the beginning of syncytium induction. First, we quantified the alterations in hormone concentrations in Arabidopsis roots infected with *H. schachtii*. Subsequently, we evaluated the expression of specific hormone and defence marker genes during the migratory and early sedentary stage of nematode infection. Further, we observed changes in nematode attraction, infection, and development in plants with altered phytohormone concentrations as well as in hormone-deficient mutant lines. Our results suggest a pivotal role of both JA and ET in the early infection of *H. schachtii*. JA seems to be a negative regulator for female development, whose signalling is suppressed after successful infection in compatible plant–nematode interaction. By contrast, ET plays a positive role in nematode attraction to the root, whereas SA is primarily involved in subsequent syncytium and female development. These findings reveal an assured involvement of various defence signalling pathways and their specificity during early CN infection in Arabidopsis.

## Materials and Methods

### Plant and nematode culture

Seeds of *Arabidopsis thaliana* L. Heynh. Columbia 0 (Col-0) and mutant lines, *dde2* (von Malek *et al*., 2002) and *lox6* (Grebner *et al*., 2013), were surface-sterilized (0.7% NaClO, 40% ethanol (EtOH)) for 8 min, washed in 70% EtOH and subsequently rinsed three times in dH_2_O. Ten seeds per dish (94 mm in diameter) were planted on modified Knop medium supplemented with 2% sucrose and subsequently grown at 16 : 8 h, light : dark at 23°C. Each 12-d-old seedling was inoculated with 50 J2s *H. schachtii* obtained from sterile stock culture (Sijmons *et al*., 1991). J2s were sterilized in 0.05% HgCl_2_ for 3 min and immediately washed three times in dH_2_O. Before inoculation, root length was categorized according to Jürgensen (2001). Inoculated plates were kept in the dark for 24 h, and subsequently transferred into a growing chamber under 16 : 8 h light : dark conditions. All experiments were repeated independently four times (*n *=* *20). Infection sites were counted at 24, 48 and 72 h after inoculation (hai), and males and females at 14 d after inoculation (dai).

### Hormone quantification

Arabidopsis plants were infected with nematodes as described earlier. Shoots and roots were collected at 24 hai, weighed, frozen in liquid nitrogen and stored at −80°C. Hormones were purified and analysed according to Dobrev & Kaminek (2002) and Dobrev & Vankova (2012). Samples (*c*. 200 mg) were homogenized and extracted with methanol : water : formic acid (15 : 4 : 1, v/v/v). The following labelled internal standards (10 pmol^−1^ sample) were added: ^13^C_6_-IAA (Cambridge Isotope Laboratories, Tewksbury, MA, USA), ^2^H_4_-SA (Sigma-Aldrich), ^2^H_2_-GA_4_, ^2^H_2_-GA_19_, ^2^H_6_-ABA, ^2^H_5_-*trans*Z, ^2^H_5_-*trans*ZR, ^2^H_5_-*trans*Z7G, ^2^H_5_-*trans*Z9G, ^2^H_5_-*trans*ZOG, ^2^H_5_-*trans*ZROG, ^2^H_5_-*trans*ZRMP, ^2^H_3_-DHZ, ^2^H_3_-DHZR, ^2^H_3_-DHZ9G, ^2^H_6_-iP, ^2^H_6_-iPR, ^2^H_6_-iP7G, ^2^H_6_-iP9G, and ^2^H_6_-iPRMP (Olchemim, Olomouc, Czech Republic). Extracts were purified using a SPE-C18 column (SepPak-C18; Waters, Milford, MA, USA) and separated on a reverse-phase cation-exchange SPE column (Oasis-MCX; Waters). The first hormone fraction was eluted with methanol (contains ABA and other acidic hormones); the second fraction, eluted with 0.35 M NH_4_OH in 70% methanol, contained cytokinin metabolites. Both fractions were separated by high-performance liquid chromatography (HPLC; Ultimate 3000, Dionex, Sunnyvale, CA, USA) and the hormones were quantified using a hybrid triple quadrupole/linear ion trap mass spectrometer (3200 Q TRAP, Applied Biosystems, Waltham, MA, USA) operated in selected reaction monitoring mode.

### Quantitative real-time (qRT) PCR

RNA was extracted from whole roots using a Qiagen RNA Plant Mini Kit according to manufacturer’s instructions including DNA digestion with DNase I (Qiagen). RNA was analysed using a Nanodrop 2000c Spectrophotometer (Peqlab, Erlangen, Germany), and cDNA synthesis was performed using SuperScriptIII reverse transcriptase (Invitrogen) according to the manufacturer’s instructions. Primers for hormone and defence marker genes are shown in Supporting Information Table S1. For the reference gene, *UBP22* (Hofmann & Grundler, 2007), the following primer sequences were used: forward, ACAACATATGACCCGTTTATCGA; reverse, TGTTTAGGCGGAACGGATACT. qPCR was performed using an ABI Prism 7300 (Applied Biosystems). The final reaction volume was 25 μl, containing 12.5 μl SYBR Green reaction kit (Invitrogen), 0.5 μl 10 nM primers, 9.5 μl ddH_2_O, and 2 μl of cDNA template. The PCR reaction was conducted in 40 cycles: 95°C for 10 min, each cycle 95°C for 15 s, 60°C for 60 s. Changes in transcript abundance were calculated using 2^−∆∆ct^ method (Schmittgen & Livak, 2008). Three independent biological replicates (pools of several individual plants) were tested in technical triplicates (averaged prior calculations).

### GeneChip

Twelve-day-old Arabidopsis plants, grown as described earlier, were infected with *H. schachtii*. Ten hours after inoculation, small root pieces containing nematodes during the migration phase performing stylet movements were cut out. Corresponding root segments from uninfected plants were used as controls. RNA was isolated using a Nucleospin RNA kit (Macherey-Nagel, Düren, Germany) according to the manufacturer’s instructions. The quality and purity of total RNA were confirmed by an Agilent 2100 Bioanalyzer (Agilent Technologies, Santa Clara, CA, USA) and a Nanodrop (Thermo Fisher Scientific, Waltham, MA, USA). The cDNA synthesis was performed with NuGEN′s Applause 3′-Amp System (NuGEN, San Carlos, CA, USA) according to the manufacturers’ instructions. NuGEN’s Encore Biotin Module (NuGEN) was used to fragment cDNA. Hybridization, washing and scanning were performed according to Affymetrix 3′ GeneChip Expression analysis technical manual (Affymetrix, Santa Clara, CA, USA). Three chips each were hybridized for control and infected samples, with each microarray representing an independent biological replicate. Primary data analysis was performed with the Affymetrix software Expression Console v1 using the MAS5 algorithm.

### Statistical analysis of microarray data

Affymetrix .CDF and .CEL files were loaded into the Windows GUI program RMAExpress (http://rmaexpress.bmbolstad.com/) for background correction, normalization (quantile) and summarization (median polish). After normalization, computed Robust Multichip Average (RMA) expression values were exported as log scale to a text file. Probe set annotations were performed by downloading Affymetrix mapping files matching array element identifiers to AGI loci from ARBC (www.arabidopsis.org). Data were analysed using *t*-test (*P *<* *0.05). The results tables include adj-*P*-values as indicators of statistical significant difference after correction for multiple testing controlling false discovery rate (Benjamini & Hochberg, 1995). Multiple tests were restricted to a subset of 62 genes involved in JA, ET and SA biosynthesis and signalling, which increases the statistical power of correction. The validation of the GeneChip data with qPCR was performed as described earlier and data analysis was done according to Siddique *et al*. (2014).

### Hormone and hormone biosynthesis inhibitor application

The following hormones or hormone donors were used: methyl-jasmonate (mJA; Sigma-Aldrich), ethephon (Eth; Sigma-Aldrich) and sodium 2-hydroxybenzoate (NaSa; Sigma-Aldrich). The following inhibitors of hormone biosynthesis were used: salicylhydroxamic acid (SHAM; Sigma-Aldrich), aminooxyacetic acid (AOA; Sigma-Aldrich) and L-2-aminooxy 3-phenylpropinoic acid (PAL-Inh; Wako-chemicals, Osaka, Japan). mJA and SA inhibitor (PAL-Inh) were dissolved in EtOH before preparing the stock solutions. All chemicals were filter-sterilized and prepared as stocks containing 0.02% Tween 20 (mJA, SHAM, Eth, NaSA, PAL-Inh as 10 mM and AOA as 25 mM). Before the experiments, different concentrations of the chemicals were tested for phytotoxicity. The final selected concentration did not result in any phenotypical changes (data not shown). Hormones and hormone inhibitors were applied to the shoots of 11-d-old plants under sterile conditions in two droplets onto two leaves per plant at the following concentrations: 60 μM mJA, 150 μM SHAM, 400 μM Eth, 15 μM AOA, 500 μM NaSa and 75 μM PAL-Inh. Distilled water containing 0.02% (v/v) Tween 20 was used as a control. After application, dishes were put back into the growing chamber.

### Nematode attraction assays

Nematode attraction assay was performed according to Dalzell *et al*. (2011). Two percent water agar plates with cylindrical counting wells (8 mm in diameter) connected via cylindrical channels (20 × 2.5 mm) were prepared. Agar discs, containing root exudates from treated and nontreated plants grown on Knop medium as described earlier, were excised from plates in close proximity to the roots. Subsequently they were put into the counting wells. One hundred J2s were placed in the middle of the connecting channel. Six plates for each treatment and each replicate were prepared and placed in the dark at room temperature. After 3.5 h, the number of J2s that reached either one or the other well was counted and classified as attracted by the root exudate of the respective agar disc. Experiments were performed in three independent replicates with six plates each (*n *=* *18). Results were calculated as the attraction rate (%) of the total number of applied nematodes.

### Statistical analysis

To test significant differences between the variants, one-way ANOVA and *t*-test (paired, for attraction assays) were performed using *post hoc* Tukey test. Statistical analysis was conducted using StatGraphics plus 4.0 software (Statpoint Technologies Inc., Warrenton, VA, USA). *P *<* *0.05 was used to determine significance.

## Results

### *Heterodera schachtii* infection triggers changes in endogenous hormone concentrations

**Figure 1:**
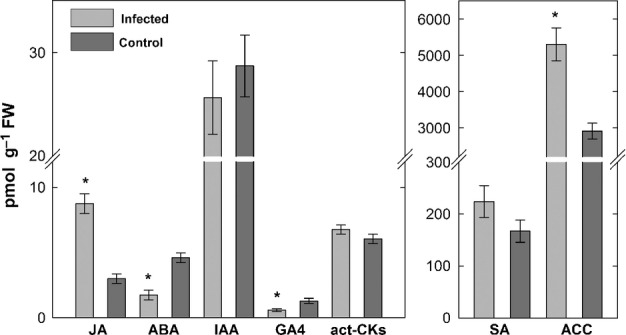
Hormone quantification (pmol g^−1^ FW) in *Arabidopsis thaliana* roots infected with *Heterodera schachtii* compared with noninfected control roots. Samples were collected at 24 h after inoculation. JA, jasmonic acid; GA4, gibberellin 4; act-CKs, active cytokinins; SA, salicylic acid; ACC, 1-aminocyclopropane-1-carboxylic acid. Values are means ± SE, *n* = 4; asterisks indicate significant differences (*, *P *< 0.05).

Hormone quantification was performed using HPLC-MS to compare nematode-infected and noninfected Arabidopsis root. Fig. 1 shows endogenous hormone concentrations at 24 hai covering nematode invasion, migration through the root tissue towards the vascular cylinder and the beginning of syncytium induction. Concentrations of JA and the immediate ET precursor, 1-aminocyclopropane-1-carboxylic acid (ACC), were highly elevated, whereas the concentrations of SA, IAA and active cytokinins (*trans*-zeatin, dihydrozeatin, isopentenyladenine, *cis*-zeatin and their ribosides, act-CKs) were unchanged in the nematode-infected root at this time point. Furthermore, concentrations of ABA and active gibberellin (GA4) were significantly reduced. The concentrations of other hormone metabolites are presented in Table S2.

### Early parasitism of *H. schachtii* triggers changes in the transcription of hormone-related genes

To correlate the results of hormone quantification with expression profiles of selected hormone and defence marker genes, time-course qRT-PCR was performed. The following transcripts were determined: *PR-5* (PATHOGENESIS RELATED 5, SA marker), *NPR-1* (SALICYLIC ACID INSENSITIVE 1, key component of SA signalling), *PDF1.2a* (PLANT DEFENSIN 1.2a, JA and ET marker, defence marker), *JAR-1* (JASMONATE RESISTANT 1, jasmonate-isoleucine synthase), *HEL* (HEVEIN LIKE PROTEIN, ET and JA marker, defence marker), and *EIN2* (ETHYLENE INSENSITIVE 2, ET signalling component). This analysis covered nematode root invasion (*c*. 0–6 hai), migration through the root tissue (*c*. 6–12 hai), syncytium induction (*c*. 12–24 hai) and development of young syncytia (*c*. 24–48 hai).

**Figure 2:**
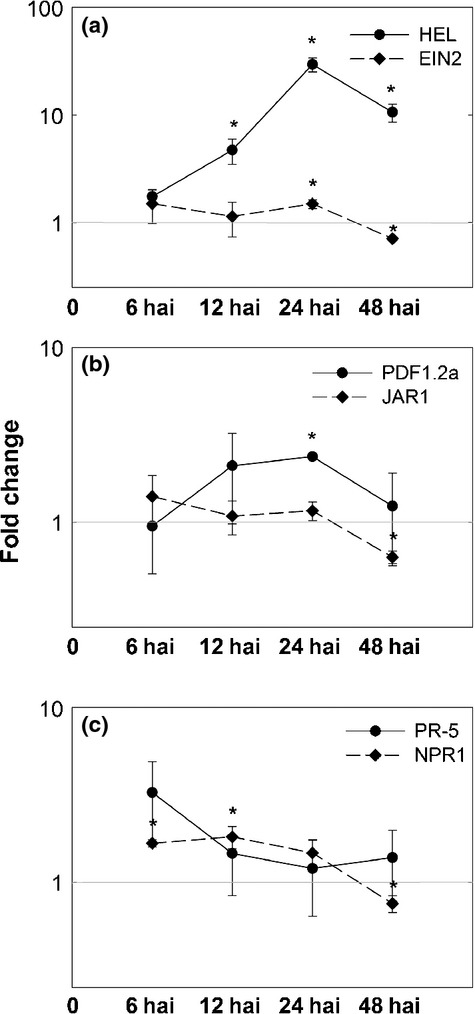
Fold changes (log_2_) of ethylene- (a), jasmonic acid- (b), and salicylic acid-related marker genes (c) in roots at 6, 12, 24 and 48 h after inoculation (hai) with *Heterodera schachtii* compared with noninfected *Arabidopsis thaliana* roots. *UBP22* was used as an internal reference. Values are means ± SE, *n* = 3, asterisks indicate significant differences (*, *P *< 0.05).

Results shown in Fig. 2 reveal significantly changed expression of *HEL*, which is up-regulated at 12 hai (4.71), subsequently culminates at 24 hai (29.42) and remains elevated until 48 hai (10.59). *EIN2* shows a first slight up-regulation at 24 hai (1.49), followed by its down-regulation at 48 hai (0.71). *PDF1.2a* is first found up-regulated at 24 hai (2.37) and its expression subsequently declines at 48 hai. *JAR1* does not show any changes in expression from 6 to 24 hai, whereas at 48 hai it is down-regulated (0.63). *PR-5* shows a slight but not significant up-regulation at 6 hai, whereas at later time points no significant change in its expression is detectable. *NPR1* is slightly up-regulated at both 6 hai (1.67) and 12 hai (1.82), and down-regulated at 48 hai (0.74).

The earlier described experiments showed clear effects of the early parasitism of *H. schachtii* on hormone concentrations, as well as expression of several hormone and defence marker genes in whole nematode-infected roots. To elucidate more specific changes in local gene expression at and around the infection area, we performed a GeneChip analysis. Root segments containing nematodes during the migratory stage at 10 hai were cut out and compared with corresponding uninfected root segments. This particular phase has been chosen, as at this time point, initial significant changes in gene expression were detected. For this study, a subset of 62 genes representing selected JA, ET and SA marker, signalling and biosynthesis genes was extracted (Table 1). To validate these GeneChip results, fold changes obtained for several genes were confirmed by qRT-PCR (Table S3). The entire GeneChip analysis will be published elsewhere (S. Siddique *et al*., unpublished).

**Table 1:** Selection of jasmonic acid- (JA), ethylene- (ET) and salicylic acid (SA)-related marker, signalling and biosynthesis genes and their fold changes obtained from a GeneChip representing migratory stage of the *Heterodera schachtii* J2s in roots of *Arabidopsis thaliana*

Function	Gene	Accession no.	Fold change	*P*-value
JA				
Marker	*PDF1.2*	At5g44420	1.1946	0.3131
	*PDF2.1*	At2g02120	−1.5691	0.2519
	*PR3*	At3g12500	2.2006	0.1199
	*PR4/HEL*	At3g04720	1.4863	0.1815
	*THI2.1*	At1g72260	−1.1404	0.1071
Signalling	*COI1*	At2g39940	−1.2203	0.0393
	*JAR1*	At2g46370	−1.3605	0.0061
	*JAZ1*	At1g19180	4.5502	0.0418
	*JAZ5*	At1g17380	6.4781	0.0454
	*JAZ6*	At1g72450	3.4011	0.0376
	*JAZ8*	At1g30135	15.8334	0.0040
	*JAZ10*	At5g13220	12.5437	0.0073
Biosynthesis	*AOC1*	At3g25760	9.7309	0.0093
	*AOC3*	At3g25780	3.3775	0.0092
	*AOC4*	At1g13280	1.2134	0.0458
	*DDE2*	At5g42650	3.1624	0.0000
	*LOX2*	At3g45140	−1.0209	0.3362
	*LOX3*	At1g17420	3.0767	0.0107
	*LOX4*	At1g72520	4.3060	0.0194
	*LOX5*	At3g22400	−1.0665	0.7320
	*LOX6*	At1g67560	2.4060	0.0041
ET				
Marker	*PDF1.2*	At5g44420	1.1946	0.3131
	*PR3*	At3g12500	2.2006	0.1199
	*PR4/HEL*	At3g04720	1.4863	0.1815
Signalling	*EIL1*	At2g27050	−1.0886	0.2343
	*EIN2*	At5g03280	−1.3594	0.0201
	*EIN3*	At3g20770	−1.0489	0.2597
	*EER4*	At1g17440	−1.0217	0.8271
	*EER5*	At2g19560	−1.1555	0.0785
	*ERF1*	At3g23240	2.7546	0.0454
	*ERF3*	At1g50640	−1.0424	0.4908
	*ERF4*	At3g15210	1.4687	0.0458
	*ERF5*	At5g47230	1.6311	0.0155
	*ERF6*	At4g17490	8.1924	0.0052
	*ERF7*	At3g20310	−1.2335	0.1230
	*ERF13*	At2g44840	5.9034	0.0444
Biosynthesis	*ACS2*	At1g01480	7.8195	0.0046
	*ACS4*	At2g22810	−1.0032	0.9700
	*ACS6*	At4g11280	4.1610	0.0595
	*ACS7*	At4g26200	1.3469	0.3159
	*ACS8*	At4g37770	1.2855	0.1568
	*ACS9/ETO3*	At3g49700	1.0784	0.2960
	*ACS11*	At4g08040	−1.0160	0.9015
	*ETO1*	At3g51770	−1.2184	0.1041
	*ETO2/ACS5*	At5g65800	−1.0555	0.2863
SA				
Marker	*PR1*	At2g14610	−1.0640	0.3459
	*PR2*	At3g57260	−1.1129	0.2464
	*PR5*	At1g75040	−1.2143	0.0464
Signalling	*EDS1*	At3g48090	−1.0742	0.3463
	*EDS5*	At4g39030	2.7249	0.0068
	*NPR1*	At4g26120	4.0646	0.0115
	*NPR3*	At5g45110	1.8615	0.0203
	*NPR4*	At4g19660	−1.1599	0.1578
	*PBS3*	At5g13320	2.4500	0.1118
Biosynthesis	*EPS1*	At5g67160	1.0281	0.7461
	*ICS1/EDS16*	At1g74710	1.3754	0.0618
	*ICS2*	At1g18870	−1.2571	0.1802
	*PAL1*	At2g37040	2.4624	0.0061
	*PAL2*	At3g53260	3.1538	0.0087
	*PAL3*	At5g04230	1.2211	0.2712
	*PAL4*	At3g10340	1.7949	0.8725

In general, most of the JA-related signalling and biosynthesis genes are up-regulated. In particular, many members of the *LOX* gene family (e.g. *LOX3*, *LOX4* and *LOX6*) show increased gene expression, which might indicate the activation of JA synthesis in roots during the migratory stage. We found up-regulation of some ET-related signalling genes, mainly from the *ERF* gene family (e.g. *ERF6* and *ERF13*). Several genes involved in ET biosynthesis, especially two members of the *ACS* gene family (*ACS2* and *ACS6*), are up-regulated. SA-related genes show only minor changes in their expression. However, some of the signalling genes and members of the *PAL* gene family (e.g. *PAL1* and *PAL2*), involved in one of the two possible SA biosynthetic pathways, are up-regulated. We could confirm the up-regulation of the SA-related signalling gene *NPR1* during the migratory stage. In the case of two signalling genes, *EIN2* and *JAR1*, GeneChip data confirmed their rather low expression levels. By contrast, ET/JA marker genes *HEL* and *PDF1.2a* are up-regulated in the whole infected root as shown by the qRT-PCR; however, according to GeneChip, their expression is not altered locally during the J2s’ migration.

### Modulation of hormone concentrations affects attraction, infection and development of *H. schachtii*

Previous experiments indicated that *H. schachtii* triggers changes in concentrations of several endogenous phytohormones in the root as well as in hormone-dependent gene expression during the early infection. Therefore, the effects of artificially altered hormone concentrations on attraction, infection and development of nematodes were tested. JA, ET and SA concentrations in the roots were modified through foliar application of exogenous hormones or by hormone biosynthesis inhibitors. Increase of hormone concentrations was achieved by application of mJA, NaSa and Eth. As, according to the GeneChip, members of the *LOX* gene family showed increased expression during the migratory stage, we used SHAM, inhibitor of lipoxygenases (LOX), the key enzymes in JA biosynthesis. Further, as members of the *ACS* family were up-regulated during the nematode migration, we used AOA, an inhibitor of aminocyclopropane-carboxylic acid synthases, rate-limiting enzymes of ET biosynthesis. There are two different biosynthetic pathways of SA. One involves isochorismate synthases (ICSs) and the other phenylalanine ammonia lyases (PALs). According to the GeneChip, some members of the *PAL* gene family were up-regulated, and hence we used PAL-Inh to specifically block the phenylpropanoid pathway responsible for one branch of SA biosynthesis, besides production of other metabolites such as lignin and flavonoids (Hahlbrock & Grisebach, 1979).

**Figure 3:**
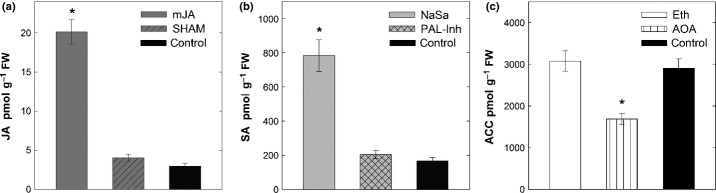
Hormone quantification using high-performance liquid chromatography-MS. Levels of jasmonic acid (JA) (a), salicylic acid (SA) (b) and 1-aminocyclopropane-1-carboxylic acid (ACC) (c) in roots of *Arabidopsis thaliana* systemically treated with the hormones methyl jasmonate (mJA), sodium salicylate (NaSa) and ethephon (Eth), and hormone inhibitors salicylhydroxamic acid (SHAM), L-2-aminooxy 3-phenylpropinoic acid (PAL-Inh) and aminooxyacetic acid (AOA). Samples were collected at 48 h after foliar application. Values are means ± SE, *n* = 4; asterisks indicate significant differences (*, *P *<* *0.05).

As a proof of concept, endogenous hormone concentrations in the roots of chemically treated and control plants were compared. Hormones were quantified 48 h after chemical application. As with the results of mJA treatment, we observed elevated concentrations of JA in roots. Similarly, NaSa treatment resulted in elevated SA concentrations. As expected, Eth did not have any effect on the concentration of ET precursor ACC, as Eth is directly converted to ET *in planta*, bypassing ACC. The ET biosynthesis inhibitor AOA affected ACC concentrations, which were significantly reduced. Inhibition of LOX and PAL by SHAM and PAL-Inh, respectively, did not impose significant effects on JA and SA concentrations. It is possible that hormone homeostasis was maintained (in the absence of infection) by conjugate hydrolysis or by activation of the alternative biosynthetic route (in the case of SA) (Fig. 3).

**Figure 4:**
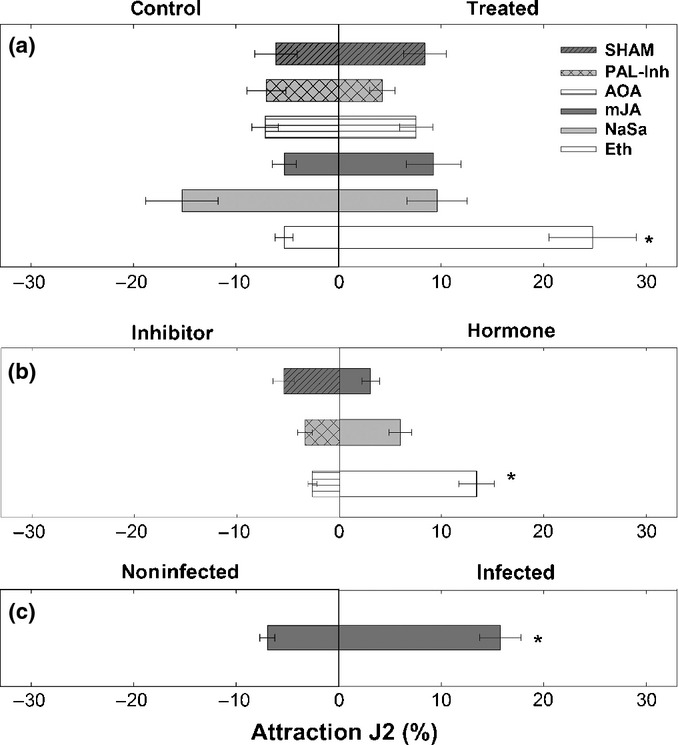
Nematode attraction assay towards root exudates obtained from control and hormone- or hormone inhibitor-treated *Arabidopsis thaliana* plants. (a) Control roots compared with hormone- and hormone inhibitor-treated plants. (b) Attractiveness of diffusates from hormone inhibitor- vs hormone-treated plants. (c) Attractiveness of diffusates from noninfected roots vs roots infected with *Heterodera schachtii*. SHAM, salicylhydroxamic acid; PAL-Inh, L-2-aminooxy 3-phenylpropinoic acid; AOA, aminooxyacetic acid; mJA, methyl jasmonate; NaSa, sodium salicylate; Eth, ethephon. Values are means ± SE, *n *=* *18; asterisks indicate significant differences (*, *P *<* *0.05).

To elucidate whether modified hormone concentrations and altered expression of hormone-related genes might change plant susceptibility towards *H. schachtii*, 24 h before inoculation exogenous hormones or their respective biosynthetic inhibitors were applied onto leaves of 11-d-old plants. Subsequently, a nematode attraction assay with agar discs containing root exudates of treated and control plants was performed. Fig. 4(a) shows that the majority of J2s were more attracted to root exudates of Eth-treated plants than to those of nontreated control plants. By contrast, mJA, NaSA, PAL-Inh, SHAM and AOA treatments did not result in major effects. In the second experiment, attractiveness of plants treated with either a hormone or its respective inhibitor was compared. Similarly to previous tests, Eth-treated plants exhibited a significantly higher attractiveness than AOA-treated plants. No differences were found between NaSa and PAL-Inh or between mJA and SHAM (Fig. 4b).

As shown in the previous experiments, *H. schachtii* triggers an elevation of JA and ACC concentrations in roots and induces an elevated expression of ET- and JA-related genes. Moreover, J2s are more attracted to root diffusates collected from Eth-treated plants. In order to investigate the effects of hormone alterations mediated by *H. schachtii* infection on further nematode attraction, we performed an additional assay using agar discs containing root exudates sampled from infected and noninfected plants. The results show that significantly more J2s moved towards the discs with root diffusates from infected plants (Fig. 4c).

**Figure 5:**
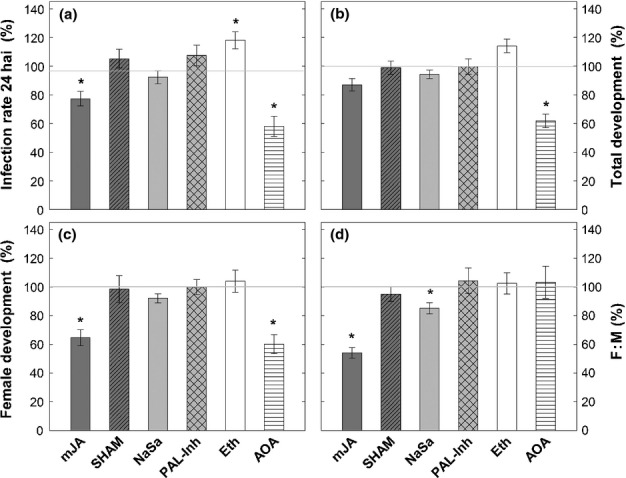
Effects of chemically modified hormone concentrations on *Heterodera schachtii* infection. (a) Infection rate at 24 h after inoculation (hai); (b) total number of female and male nematodes at 14 d after inoculation (dai); (c) number of females at 15 dai; (d) female : male (F : M) ratio. Hormones and inhibitors were applied 24 h before nematode inoculation. Values are mean deviances from control (= 100%) ± SE, *n *=* *20; asterisks indicate significant differences (*, *P *<* *0.05). mJA, methyl jasmonate; SHAM, salicylhydroxamic acid; NaSa, sodium salicylate; PAL-Inh, L-2-aminooxy 3-phenylpropinoic acid; Eth, ethephon; AOA, aminooxyacetic acid.

Subsequently, an infection assay was conducted to test whether alterations in endogenous hormone concentrations affect the infection and development of *H. schachtii*. Infection sites were monitored at three time points: 24, 48 and 72 hai. Males and females were counted at 14 dai and the female : male (F : M) ratio was calculated. This parameter is a meaningful indicator of the developmental conditions provided by the host. It is known that more females than males can develop under optimal nutritional and environmental settings (Triantaphyllou, 1973). Eth treatment is beneficial for nematodes, which infect more quickly (with a *c*. 20% higher infection rate at 24 hai) when compared with control plants (Fig. 5a). This effect subsequently declines, resulting in no difference in the number of infection sites at 3 dai (data not shown). No significant changes in male and female development (Fig. 5b,c) or in F : M ratio (Fig. 5d) were detected for Eth-treated plants.

Lower concentrations of ACC triggered by AOA treatment resulted in reduced numbers of J2s infecting the roots (Fig. 5a). Accordingly, the counts of female and male nematodes were significantly decreased (Fig. 5b,c); however, there was no effect on F : M ratio (Fig. 5d). After foliar application of mJA, infection rates were lower compared with nontreated plants during the first 24 hai (Fig. 5a). Although the total number of developing nematodes was not altered (males and females together; Fig. 5b), the female counts were significantly reduced (Fig. 5c), leading to decreased F : M ratio (46% lower compared with controls; Fig. 5d). No differences in nematode infection were observed in plants treated with the JA inhibitor SHAM. NaSa treatment, although proven to elevate SA concentrations in roots (Fig. 3b), did not show any significant effects on infection and development of nematodes, except for a significantly reduced F : M ratio (15% lower compared with controls; Fig. 5d). PAL-Inh did not show any impact on *H. schachtii* development.

### Impaired JA biosynthesis has an effect on *H. schachtii* development

**Figure 6:**
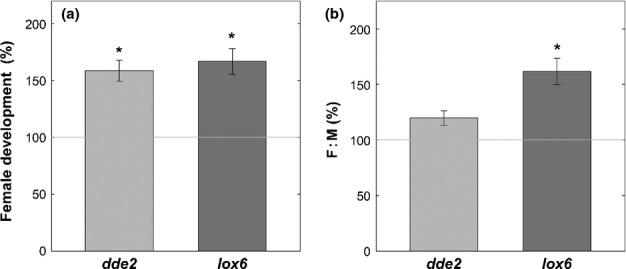
*Heterodera schachtii* infection assay with *Arabidopsis thaliana* mutants defective in jasmonic acid biosynthesis, *dde2* and *lox6*, compared with wild-type plants. (a) Female development at 14 d after inoculation; (b) female : male (F : M) ratio. Values are mean female development per plant ± SE, *n *=* *70; asterisks indicate significant difference (*, *P *<* *0.05).

Experiments with hormone biosynthesis inhibitors, SHAM, PAL-Inh and AOA, revealed that only AOA significantly affected nematode development. Concerning JA-related mutants and their impact on nematodes, only limited information is available. The foliar SHAM application did not change the JA concentrations in the root and did not trigger any effects on nematodes. Taking this into account we decided to analyse nematode development on JA biosynthesis mutants: *dde2* (allene oxide synthase, a key enzyme in JA biosynthesis) and *lox6* (lipoxygenase involved in the early steps of JA biosynthesis in plastids). As shown in Fig. 6, in roots of both mutant lines, a significantly greater number of females could develop (Fig. 6a) and the F : M ratio in the *lox6* mutant was significantly higher in comparison with the wild-type (Fig. 6b).

## Discussion

Sedentary plant-parasitic nematodes comprise a large group of important biotrophic endoparasites. In contrast to foliar pathogens, there is considerably less knowledge concerning signalling pathways involved in plant defence effective against these root parasites (Wubben *et al*., 2008). In mature feeding sites, the plant defence responses are generally suppressed (Szakasits *et al*., 2009), whereas at the onset of the parasitism they are induced (Puthoff *et al*., 2003; Alkharouf *et al*., 2006; Ithal *et al*., 2007; Mazarei *et al*., 2011). Hence, these early defence mechanisms are critical and can decide about success or failure of the nematode. However, there is still only limited information on how nematodes challenge plant stress responses at the onset of infection and which mechanisms at later stages lead to the impairment of plant defence. Phytohormones have been proposed to be involved in these processes (reviewed in Goverse & Bird, 2011); however, their exact role during juveniles’ root attraction, penetration, migration and initiation of syncytium formation has been studied only to a minor extent. Therefore, the aim of this study was a broad analysis of possible functions of the main stress-related phytohormones during attraction and early parasitism of *H. schachtii* in Arabidopsis.

### JA triggers early defence responses against *H. schachtii*

We showed that endogenous JA concentrations are strongly elevated during early nematode parasitism, which is additionally confirmed by the significant up-regulation of several genes associated with JA biosynthesis. In addition, our expression studies revealed an up-regulation of some JA/ET marker genes such as *PR-3* and *PR-4* (*HEL*) during the migration stage. However, Hamamouch *et al*. (2011) did not observe any changes in their expression at later stages of *H. schachtii* infection (5 dai and later). Interestingly, the same authors showed an elevated expression of *PR-3* in roots infected with RKN. Ithal *et al*. (2007) performed a study of the CN *H. glycines* on soybean and, in contrast to our results for the migratory stage (6–12 hai), observed a down-regulation of genes encoding JA biosynthetic enzymes in developing syncytia. The authors concluded that an active suppression of JA biosynthesis and signalling plays an important role during syncytium formation and thus nematode development. Similarly, Ji *et al*. (2013) also detected suppression of JA biosynthesis in 7 and 14 dai giant cells of RKN. On the other hand, RKN susceptibility of tomato does not depend on JA biosynthesis but requires an intact JA signalling pathway via *Coi-1* (Bhattarai *et al*., 2008). These differences in the activation of JA-dependent pathways are intriguing. They are a result not only of the different plant species used but also of the very distinct way of infecting the host root by CNs and RKNs. Infective juveniles of RKNs cause much less tissue damage as a result of intercellular migration within the root in comparison to intracellular invasion of CNs. This could explain the different roles of JA in signalling during early parasitism of both genera in different hosts.

Further, we showed that artificially elevated JA concentrations in roots led to a reduction, whereas JA biosynthesis mutants led to an enhancement, of female development. Similar to these results, it was shown that exogenous JA treatments of spinach, oat and tomato increased resistance against different nematode species (Thurau *et al*., 2003; Soriano *et al*., 2004; Cooper *et al*., 2005). The JA pathway was also found to play a significant role in defence against the RKN *Meloidogyne graminicola* in rice, while mJA application reduced gall number and JA-deficient mutant plants were more susceptible (Nahar *et al*., 2011).

Here, we demonstrate that the invaded plant produces elevated amounts of JA during nematode migration to defend itself, which is supported by an up-regulation of several JA biosynthetic genes, such as *LOX* and *AOS*. At the same time, the nematode attempts to switch off the JA-based plant defence responses by triggering the up-regulation of genes that are known to suppress plant JA signalling, such as *JAZ8* (Shyu *et al*., 2012), as well as the repression of JA signalling via Coi1 and Jar1. In the later stages, as a result of the down-regulation of, for example, LOX genes, the JA biosynthesis is supressed in soy plants infected with *H. glycines* (Ithal *et al*., 2007). Based on these results, we suggest that in compatible interactions at the onset of CN infection, the nematode is able to supress JA-dependent plant defence. This is supported by the fact that the artificial elevation of JA concentrations interferes with nematodes’ ability to suppress JA biosynthesis and signalling, resulting in a significantly decreased number of developing females. On the other hand, to elucidate the effects of decreased JA concentrations on *H. schachtii*, we performed an infection assay with two mutants deficient in JA biosynthesis, *dde2* (AOS) and *lox6*. As expected, the results revealed an increased female development on both mutants in comparison to the wild-type. In addition, *lox6* showed a significantly enhanced F : M ratio. Our results are in line with findings recently published by Ozalvo *et al*. (2014) showing another lipoxygenase mutant (*LOX4*) to be similarly more susceptible to *H. schachtii*. We conclude that in compatible plant–nematode interactions, JA is a main player during early plant defence; however, the nematode contrived to suppress and overcome JA-related defence responses and successfully infect the host.

### ET plays a pivotal and positive role in nematode attraction

The existing data concerning the role of ET in nematode defence and development are complex and often contradictory, very likely because of its pleiotropic role in defence and development, where ET seems to play different roles at different stages of nematode infection (reviewed in Kyndt *et al*., 2013). For instance, Wubben *et al*. (2001) found root exudates of ET-overproducing mutants to be more attractive to J2s. Similarly, in our attraction assays, Eth-treated plants were more attractive and could be infected much faster, resulting in a higher infection rate. Thus, the high attractiveness of roots with elevated ACC/ET concentrations towards infective J2s suggests a pivotal role of the ET pathway during the host-finding process. Moreover, in preinfected plants, this indirect effect might be a result of altered plant defence as well as significantly elevated ET concentrations, which are perceived by following juveniles as clear evidence that the roots are already successfully infected and therefore that the plant is most probably a suitable host. In contrast to CN, Fudali *et al*. (2013) found that Arabidopsis roots treated with an ET inhibitor are more attractive, whereas ET-overproducing mutants are less attractive to juveniles of the RKN *Meloidogyne hapla*. These disparities might be the result of a differing experimental setup (‘medium plugs’ vs living roots, respectively), and the fact that CNs have a narrow host range and probably respond to a different set of factors responsible for the attraction to host roots than RKNs do (Fudali *et al*., 2013).

Interestingly, although ET plays a positive role in CN attraction, at later stages its suppression in syncytia indicates a rather negative function for nematode development, which is supported by studies of Mazarei *et al*. (2003) and Ali *et al*. (2013). The ethylene-responsive element binding protein gene GmEREBP1 was found to be down-regulated in susceptible soybean infected with CN, whereas it is up-regulated in resistant roots (Mazarei *et al*., 2003). Similarly, it was shown that the ET-responsive RAP2.6 gene is down-regulated in syncytia and its overexpression leads to enhanced resistance, which the authors suggest is a result of the activated JA pathway and callose deposition (Ali *et al*., 2013).

On the other hand, older studies by Glazer *et al*. (1983, 1985) showed, for RKN, that enhanced ET concentrations support giant cell enlargement, whereas chemical blocking of ET inhibits their development. Similarly, for CNs, Goverse *et al*. (2000) and Wubben *et al*. (2001, 2004) found evidence that an intact ET pathway is required for successful root colonization and syncytial development. The authors showed that ET-insensitive Arabidopsis mutants as well as the application of an ET inhibitor led to a significant decrease in the number of *H. schachtii* females, similar to our results after AOA application. Moreover, ET-overproducing mutants and the application of ACC increased female development (Wubben *et al*., 2001, 2004). The authors suggest that the hypersusceptibility could be partially attributed to the increased juvenile attraction, rather than to better developmental conditions. After Eth treatment, we did not observe any differences in female development, which could be a result of the application method used and the following time-dependent attenuation of the effect.

Based on our results and previous studies, we conclude that the role of ET is strongly dependent on the stage of parasitism, shifting from a positive role in attraction and root invasion to a negative role in further nematode development.

### SA acts as a negative regulator during syncytium and female development

It is generally accepted that the SA pathway is predominantly effective against biotrophic pathogens (Pieterse *et al*., 2009). Wubben *et al*. (2008) showed that SA marker genes *PR-2* and *PR-5* are induced in infected roots from 3 dai on. *PR-1* expression as well as endogenous SA concentration, however, remained unaltered. Hamamouch *et al*. (2011) found up-regulation of *PR-1*, *PR-2* and *PR-5* in roots infected with *H. schachtii* at 5 dai and at later time points. Here, we show that neither the concentrations of endogenous SA in nematode-infected roots at 24 hai nor the transcript abundance of the SA marker gene *PR-5* from 6 hai on were significantly altered. As shown by Wubben *et al*. (2008) on mutants defective in SA accumulation or signalling (*sid2-1*, *pad4-1* and *NahG*), a significantly greatern number of *H. schachii* females could develop. To test whether elevated SA concentrations affect host susceptibility during early infection, we performed nematode attraction and infection assays on NaSa-treated plants. This treatment, similar to the results of Wubben *et al*. (2008), triggered a lower F : M ratio but did not cause any other effects on *H. schachtii* attraction and infection rates. In the case of Pal-Inh, blocking one of the two SA biosynthetic pathways as well as other metabolites produced by PAL, for example lignin, we would expect an increase in plant defence against CN. This treatment, however, did not show any significant effects on nematodes. Therefore, we speculate that either PAL does not play an eminent role during early infection of *H. schachtii* or our experimental setup was not optimal. Foliar application of NaSa and PAL-Inh might trigger only short transient effects in roots. The use of mutant lines or growth medium directly supplemented with the chemicals ensuring their constant delivery to the root could result in significant changes in nematode susceptibility.

Taken together, these results suggest that well-established parasitism of CN requires a local suppression of SA signalling and that SA does not play a major role during early *H. schachtii* infection but rather acts as a negative regulator during later phases of parasitism, when syncytium matures and nematodes differentiate sexually.

### The role of other stress-related hormones during early nematode infection

In addition to the three main stress hormones, ABA, GA, auxins and cytokinins play pivotal roles in fine-tuning of hormone-based signalling in plants, for instance as modulators of the SA/JA signalling backbone (Pieterse *et al*., 2012). Here, we show that concentrations of ABA, together with its catabolites, as well as the IAA precursor IAN and oxidated IAA, active GAs and cytokinins were decreased in nematode-infected roots compared with control roots. Generally**,** ABA is associated with responses to abiotic stresses (Cutler *et al*., 2010), but it has also recently been suggested to act as an important fine-tuning regulator of defence responses in various plant–pathogen interactions (Mauch-Mani & Mauch, 2005; Studham & MacIntosh, 2012). ABA is known to counteract the SA and ET/JA basal defence and to suppress ET action after pathogen infection. Nahar *et al*. (2012) described the negative role of ABA in rice defence against the migratory nematode *Hirschmanniella oryzae*, while Karimi *et al*. (1995) found a lower reproduction rate of *Meloidogyne incognita* on ABA-treated potato plants. Our results suggest that reduced ABA concentrations in *H. schachtii*-infected Arabidopsis roots might result from the ABA/ET counteraction.

Gibberellins are known to regulate many developmental processes throughout the plant’s life (De Bruyne *et al*., 2014), amongst others, plant immunity, where they allow appropriate modulation of defence responses (Denance *et al*., 2013; De Bruyne *et al*., 2014). Accordingly, Kyndt *et al*. (2012) found strongly induced GA concentrations in rice infected with the RKN *M. graminicola* at 3 dai, and Klink *et al*. (2007) detected an up-regulation of GA biosynthesis genes in soybean infected with *H. glycines*. By contrast, in our system at an earlier time point, GA concentrations are significantly reduced, which is in line with findings by Bar-Or *et al*. (2005) and Puthoff *et al*. (2003) showing genes involved in GA deactivation to be up-regulated in RKN- and CN-infected plants. These rather fragmentary findings show that the putative role of GA in defence against nematodes in different plant systems varies and needs to be studied in more detail.

Similarly, auxins and cytokinins are known to function in regulation of plant growth and in response to abiotic stresses, and have recently emerged as key players in plant–pathogen interactions (Mauch-Mani & Mauch, 2005; Kazan & Manners, 2009; Fu & Wang, 2011). In the case of CNs (Goverse *et al*., 2000; Grunewald *et al*., 2009) and RKNs (Karczmarek *et al*., 2004; Kyndt *et al*., 2012), it was shown that feeding site initiation and development are auxin-dependent. Here, we found slightly reduced concentrations of IAA and a significantly reduced concentration of the IAA precursor IAN at the very early phase of *H. schachtii* parasitism. In accordance with many reports, we propose that auxin does not play a central role during early defence, but rather later in syncytium induction and development. Cytokinins, which act in combination with auxin, are suppressed by the RKN *M. graminicola* infection at 3 dai, whereas at later developmental stages, their levels are induced (Lohar *et al*., 2004; Kyndt *et al*., 2012). Kyndt *et al*. (2012) postulated that changes in cytokinin concentrations caused by RKNs could be important for swelling of the root meristem and might additionally be necessary for the conversion of galls into nutrient sinks. As, at 24 hai, we also found reduced concentrations of cytokinins, we propose that they play a rather minor role in defence, but that their function in *H. schachtii* infection might be similar to their role in galls induced by RKNs.

The presented results clearly show that JA does play an important role during early plant defence against *H. schachtii*. However, in compatible interactions, the parasite is able to suppress and overcome JA-related defence responses and successfully infect the host. For ET, we conclude that its role is strongly dependent on the stage of parasitism. Elevated ET concentrations in roots are highly attractive to the nematode; however, existing data suggest its negative role in further nematode development. Further, CN establishment requires local suppression of SA signalling, whereas SA does not play a major role during early *H. schachtii* attraction and infection, but rather acts as a negative regulator during later phases of parasitism. Taken together, our results indicate that nematodes are able to trigger sophisticated changes in hormone biosynthetic and signalling pathways in strict dependence on their parasitism stage.

## Supporting Information

Table S1 Primers for hormone and defence marker genes used in quantitative real-time (qRT) PCR **Table S2** Hormone quantification in *Arabidopsis thaliana* roots infected with *Heterodera schachtii* in comparison with noninfected roots **Table S3** Validation of GeneChip data by quantitative real-time (qRT) PCR
