# Supplementary material for: Role of stress-related hormones in plant defence during early infection of the cyst nematode Heterodera schachtii in Arabidopsis
Source: New Phytol. 2015 Mar 30;207(3):778–89. doi: 10.1111/nph.13395 (PMC4657489; doi:10.1111/nph.13395)
Supplement: Table S1 — Primers for hormone and defence marker genes used in quantitative real-time (qRT) PCR Table S2 Hormone quantification in Arabidopsis thaliana roots infected with Heterodera schachtii in comparison with noninfected roots Table S3 Validation of GeneChip data by quantitative real-time (qRT) PCR [file nph0207-0778-sd1.pdf]

Supporting Information Tables S1–S3

Article title: Role of stress-related hormones in plant defence during early infection of the cyst nematode *Heterodera schachtii* in Arabidopsis

Authors: Nina Kammerhofer, Zoran Radakovic, Jully M. A. Regis, Petre Dobrev, Radomira Vankova, Florian M. W. Grundler, Shahid Siddique, Julia Hofmann and Krzysztof Wieczorek

Article acceptance date: 3 March 2015

The following Supporting Information is available for this article:

Table S1 Primers for hormone- and defence marker genes used in quantitative real-time (qRT)-PCR

Table S2 Hormone quantification in *Arabidopsis thaliana* roots infected with *Heterodera schachtii* in comparison to noninfected roots

Table S3 Validation of GeneChip data by quantitative real-time (qRT)-PCR

Table S1 Primers for hormone- and defence marker genes used in quantitative real-time (qRT)-PCR

Gene	Accession no.	Primer sequence
ACS2	At1g01480	F: GGATGGTTTAGGATTTGCTTTG<br>R: GCACTCTTGTTCTGGATTACCTG
ACS6	At4g11180	F: GTTCCAACCCCTTATTATCC<br>R: CCGTAATCTTGAACCCATTA
AOC1	At3g25760	F: CTTAAGCCCAGTGGAGTTGTAAG<br>R: TATACAGGACACGAGAAAGATAAGAC
DDE2	At5g42650	F: CGGGCGGGTCATCAAGTT<br>R: GCTCCCATCGTGAGTTCTCC
EIN2	At5g03280	F: CCTTGTCACTAATGGAGCAGG<br>R: CACGATGAAGCCAAGCG
ERF13	At2g44840	F: CGGCGTTAACTCACGGATGT<br>R: ACGGCAAGTCGCTCCAGTTAT
ERF6	At4g17490	F: GAAAACCGCCGTTGAAGATC<br>R: CGGTTGCGAATTGAATCCA
HEL	At3g04720	F: GATAAGCCGTACGCATGGC<br>R: TCACCCTTAAACACTTGCCG
JAR1	At2g46370	F: GCTACATTTGCTGTGATTCCG<br>R: GGTATCGATACAACCCTGCG
JAZ10	At5g13220	F: TCGCAAGGAGAAAGTCACTGCAAC<br>R: CGATTTAGCAACGACGAAGAAGGC
JAZ8	At1g30135	F: TGTGTTTTTCTTCAGATGTTACCC<br>R: TCTCTGCTTGCGATCGATATT
LOX3	AT1g17420	F: CGGATAGAGAAAGAGATTGAGAAAAGGAAC<br>R: AGGTACACCTCTACACGTAACACCAGGC
LOX4	At1g72520	F: TCGCTAACTTTGGTGAGATCGATAG<br>R: TCGTCATCTCGAAGCCATGCATATT
LOX6	At1g64560	F: CCTCATGAGAGAATTTATGACTACG<br>R: TGCTCTGAAGGTATCTCTCTTGATT
NPR1	At1g64280	F: GAGTTGCACTTGCTCAACGTC<br>R: GCTATCTTTACACCCGGTGATG
PAL1	At2g37040	F: TGTAGCGCAACGTACC<br>R: GTTCGGGATAGCCGATG
PAL2	At3g53260	F: CACTGCTATGTGTGAAGGTAAACTT<br>R: CAGCAAAATTAAAATTGAAACAAAA
PDF1.2	At5g44420	F: TGTTCTCTTTGCTGCTTTCG<br>R: TTTCCGCAAACCCCTGAC
PR5	At1g75040	F: AGGCTGCAACTTTGACGC<br>R: AGAAATCTTTGCCGCCATC

Table S2 Hormone quantification in Arabidopsis roots infected with *Heterodera schachtii* in comparison with noninfected roots

	Infected (pmol g^{-1} FW)	SE	Non infected (pmol g^{-1} FW)	SE	*P*-value
**ABA**	1.72	0.38	4.59	0.37	0.0016
**DPA**	12.11	1.21	17.20	1.26	0.0272
**PA**	0.30	0.20	1.10	0.25	0.0467
**ABA-GE**	5.53	0.44	7.73	1.02	0.0945
**NeoPA**	0.07	0.05	0.08	0.02	0.9430
**IAA**	25.64	3.53	28.69	2.97	0.5339
**IAA-Asp**	2.25	0.63	2.72	0.29	0.5221
**OxIAA**	147.79	9.53	119.01	7.71	0.0573
**Ox-IAA-GE**	62.92	8.42	145.61	14.02	0.0023
**PAA**	134.85	10.85	162.65	8.17	0.0866
**IPyA**	31.35	4.23	28.84	3.43	0.6610
**IAN**	402.99	63.32	634.05	56.03	0.0341
**SA**	224.00	30.55	166.98	21.34	0.1769
**JA**	8.75	0.76	2.98	0.37	0.0005
**JA-Ileu**	0.22	0.05	0.07	0.05	0.0970
**cis-OPDA**	9.46	1.32	7.74	0.55	0.2735
**GA4**	0.57	0.12	1.27	0.20	0.0225
**GA8**	0.54	0.10	0.43	0.14	0.5433
**GA19**	1.12	0.15	1.25	0.32	0.7226
**act CK**	6.77	0.36	6.05	0.36	0.2079
**CK N-glc**	8.93	0.38	13.43	0.43	0.0002
**CK O-glc**	2.01	0.47	2.46	0.38	0.4850
**CK P**	1.06	0.09	1.91	0.14	0.0028
**cZ der**	3.92	0.19	6.07	0.23	0.0004
**ACC**	5301.05	451.06	2908.02	222.77	0.0031

ABA, abscicic acid; DPA, dihydrophaseic acid; PA, phaseic acid; ABA-GE, abscisic acid glucosyl ester; NeoPA, neophaseic acid; IAA, Indole-3-acetic acid; IAA-Asp, indole-3-acetyl-aspartate; OxIAA, 2-oxindole-3-acetate; PAA, 2-phenylacetic acid, IPya, indole-3-pyruvic acid; IAN, indole-3-acetonitrile; SA, salicylic acid; JA, jasmonic acid; JA-Ileu, jasmonic acid isoleucine; cis-OPDA, 12-oxophytodienoic acid; GA, gibberellin; CK, cytokinins; cZ, zeatin; ACC, 1-aminocyclopropane-1-carboxylic acid.

Table S3 Validation of GeneChip data by quantitative real-time (qRT)-PCR

Gene	Accession no.	GeneChip data (Fold change)	qRT-PCR (Fold change)
ACS2	At1g01480	7.81	1.36
AOC1	At3g25760	9.73	∞
JAR1	At2g46370	-1.36	-2.97
JAZ10	At5g13220	12.54	29.5
DDE2	At5g42650	3.16	4.31
LOX3	At1g17420	3.07	38.74
LOX4	At1g72520	4.30	3.04
PR4/HEL	At3g04720	1.48	1.64
EIN2	At5g03280	-1.35	-4.43
ERF13	At2g44840	5.90	8.33
PR5	At1g75040	-1.21	-3.29
PDF1.2	At5g44420	1.19	∞
NPR1	At4g26120	4.06	2.83

Transcript abundances were measured in *Arabidopsis thaliana* root segments containing *Heterodera schachtii* juveniles at the migratory stage and compared with control root segments used for GeneChip. ∞, mRNA was detected in infected but not in control root, making it impossible to calculate a fold change value.
